# Supplementary material for: Chromothripsis during telomere crisis is independent of NHEJ, and consistent with a replicative origin
Source: Genome Res. 2019 May;29(5):737–49. doi: 10.1101/gr.240705.118 (PMC6499312; doi:10.1101/gr.240705.118)
Supplement: Supplemental Material [file supp_gr.240705.118_Supplemental_file_1.zip › contigs/annotated_contigs/DB73/contig.6.DB73_length_380_mean_cov_5.52631578947.docx]

**DB73_length_380_mean_cov_5.52631578947**

```
TCACATTACTGCCTGAACTTCACCTCCTGGCAGATTGCAGCGGTATTGGATTCTCATAGGAGCGTGAACCCTATTGTGAACTGCACATG
 >chr3:188352357-188352461 + E=4e-48
CGAGGGATATATTT|T|ATTACATATTATATAATTTATATAATAATATAT|ATATTATTTTA|TAATTTTTTAAATTATATATATATAT
               >chr3:96339266-96339300 - E=6e-03 p=0e+00    >chr9:75931824-75931863 +
AATTATATATATATA|ATTAAAATTTTTTAAATTATATATAAT|TT|ATATATATAAATATATATATTTTATATTTATAT|TACAA|TT
 E=4e-07 p=0e+00  >chr7:42152223-42152250 + E=9e-02 p=0e+00  >chr2:7733891-7733924 -
ACTTTTCTCCCTGGCTTTTGGTTCACTTTTTGTACTTTTAACAAGTGGGTGAATTATTTGATAATTTTGAGGAAGATTATTCTTTTAAA
 E=2e-02  >chr3:190128212-190128336 + E=3e-63
TTCAAACTAGTATGTCAATGCCTACCATTACTC
```
